# New Government Grants

**Published:** 1914-10-03

**Authors:** 


### NATIONAL INSURANCE ACT DEVELOPMENTS.

### New Government Grants.

In normal times the grant of more than one million pounds by the Government to the national health service would attract considerable attention, but under the very extraordinary conditions which prevailed on August 4, when the Civil Service Supplementary Estimates were considered by the House of Commons, no less than £1,086,500 was voted for the purpose without comment. But because the House of Commons in the special circumstances did not discuss the proposals necessarily involved in regulating this expenditure is no reason why the grants should not be critically examined by the public generally.

The whole of the circumstances in which the grants have come to be made in their present form are somewhat remarkable. In the first place an announcement was made by the Chancellor of the Exchequer in his Budget speech that it was intended to set aside one million pounds as a supplementary grant for national health insurance over and above the statutory requirements under the Acts. No details were given, however, and it was stated that fresh legislation in the form of another Amendment Act would be required to give effect to the proposals of the Government. Owing to the highly contentious character of the legislative programme the proposed Bill did not even reach the initial stage of presentation, and the original project had to be abandoned at least for the time being. The process that has now been adopted was to authorise grants for specific purposes and to entrust the departments charged with the administration of the funds to draw up regulations for their disposal.

### Safeguards for Proper Use of the Funds.

From some points of view it may be an advantage that the expert authorities who are responsible for the actual distribution of the funds should also, from the fullness of their knowledge, have the opportunity of determining the basis of that distribution.

Nevertheless the process adopted is open to serious criticism, because it is virtually an impossibility for any executive body, with only a limited fund for disposal, to satisfy the demands of all those who consider that they have a claim thereupon; and the regulations, however well they may be drawn, lack the authority of an Act of Parliament even though specific powers are conferred on the Government department to frame such regulations. Furthermore, it is a recent departure in public finance, and is at variance with the established rule that those who provide the money should regulate how it is to be spent.

It is, however, a compliment to the Joint Committee of the Insurance Commissioners, and to the other authorities involved, that they have been entrusted with the very important tasks that have been delegated to them. We have no reason to doubt that the intentions of the Government will be satisfied to the full, and until the contrary is proved we shall believe that the very best use is being made of the money that has now been set aside for the betterment of the administration of the national health services, and for their extension in several important directions. It is an added safeguard in the case of the grants directly made for national insurance purposes that the schemes for distribution of the funds are to be framed by the Joint Committee in consultation with the Commissions for the several parts of the United Kingdom, while in all cases the final adoption of the schemes is subject to the approval of the Treasury.

### The Grants Summarised.

Coming to details, it may be remarked that the only particulars available at the time of writing are contained in a House of Commons paper (398) dated July 28, and the grants fall under three main heads.

Taking first the grants made directly for the purpose of national health insurance to the Joint Committee of the Commissioners, it is to be noted that the power to make the grants is contained in Section 1 of the National Insurance Act of 1913.

The following is a summary of funds placed at the disposal of the Joint Committee for the purposes named:—

| | | £ |
|---|---|---|
| *a.* | Administration of Medical Benefit, &c. | 116,500 |
| *b.* | Arrears of Contributions ... ... | 80,000 |
| *c.* | Sickness Benefit (Women) ... ... | 500,000 |
| *d.* | Medical Referee-Consultants, &c. ... | 50,000 |
| *e.* | Supplementary Medical Services ... | 50,000 |
| *f.* | Nursing Grants ... ... ... ... | 100,000 |
| *g.* | Sanatorium Benefit ... ... ... | 100,000 |
| *h.* | Medical Attendance, etc., of aged and disabled members of Friendly Societies | 28,000 |
| | Total ... | £1,024,500 |

The various items above mentioned call for some comment on the lines of the official description of the purposes for which the grants are made.

### Provision for Health Lectures.

It has been found by experience that the basis previously adopted for meeting the administrative expenses of the Insurance Committees was not entirely satisfactory, and in consequence provision has been made under heading *a* for an additional sum whereby the basis may be modified to meet the known requirements. In this respect, however, the estimate is somewhat misleading as a guide to future years, because the administration accounts of several of the Insurance Committees had not reached the amount estimated, and in consequence there was a balance in the hands of the Commisioners that could be set against the increased demands of the new scheme in the current year. On the other hand, an additional sum of £15,000 is included in the estimate from which to liquidate the deficiencies in this account which have been incurred by certain other Insurance Committees. But more interesting, in a general way, is the fact that under this heading is included a sum of £20,000 to be distributed in grants to Insurance Committees towards the cost of health lectures and the publication of information on questions of health. Provision was made in the principal Act for this wider aspect of the functions of Insurance Committees, but as no funds were specifically allotted to them for the purpose and their resources were only sufficient to meet more urgent requirements no action of any consequence had been taken in this direction. It is to be noted that the grant is made for the year only, and is in no way binding upon the Government as an annual charge, though any balance unexpended at the end of the year is not to revert to the Exchequer, but is to be carried forward in future estimates. In view of the present unsettled conditions, which naturally tend to hamper new enterprise, it may be some time before the lectures are organised; but it may be hoped that when they are undertaken they will be made thoroughly worthy of the cause they are intended to serve.

### Grants in Aid of Arrears and to Women's Societies.

The grants in aid towards arrears of contributions and sickness benefit (women)—namely, *b* and *c* in the above list—fall into an entirely different category. In both cases the funds are to be actually distributed by approved societies. The former is a purely benevolent grant made for the purpose of mitigating the penalty of reduced rates of benefit which apply when insured persons are in arrear. So far the only information available is that the fund is to be restricted for the benefit of those members who, while out of work, have incurred "penalty arrears" exceeding six weeks in the case of men and five weeks in the case of women. It may be remarked that in order that this grant may become operative during the next "penalty year," commencing in November, it will be necessary for regulations to be drafted without delay, as the period in which "penalty arrears" may be paid expires in the first week in October. All those who at present have arrears charged against them exceeding six or five weeks respectively for men and women, and who require assistance to reduce such arrears, should forthwith apply to their societies for information in the matter. It appears from the extent of the grant that the Government is anxious to prevent any serious curtailment in the normal rates of benefit on account of arrears of contribution due to unemployment.

The grant of £500,000 towards the cost of sickness benefit, including loss of contributions, incurred since the commencement of the Act by approved societies having women members, is the largest item in the list. It is intended, by means of this grant, to defray as far as possible the expected deficiencies in the funds of approved societies which have women members, and thus to obviate the necessity for a reduction of benefits when the first valuations come to be made next year. The method by which the grant is to be distributed to the societies—namely, on a basis calculated with reference to the relative incidence of incapacity during pregnancy among the members—is a tacit admission that the excess sickness claims are, in the main, due to pregnancy. Handsome as the grant may appear in amount it is at best but a palliative, for unless the fundamental relation between rates of benefit and contribution are revised, or unless the grant is to be of a permanent character, the deficiencies in women's societies will constantly recur, and a method of permanently meeting the difficulty must eventually be found, and the sooner the better.

### Grants Towards Adequate Medical Treatment.

The grants included under headings *d*, *e*, and *f* above are particularly interesting, as they indicate that the Government is now satisfied that the services already provided under the panel system are not adequate medical attendance in the sense that specialist services have not up till now been available. The grant for medical referee-consultants is to be administered by the Joint Committee of the Insurance Commissioners themselves, and the money is to be used for the remuneration of referees and consultants, and in the payment of travelling expenses of insured persons presenting themselves for examination. The institution of this service should have a beneficial effect in reducing malingering. It is apparently intended to charge certain fees from approved societies making use of the service, for it is stated that such payments are to be made into the Exchequer. Hospital managers will, doubtless, note that the grant for supplementary medical services is designed to meet the cost of providing specialist consultations in connection with the treatment of insured persons, and to aid the equipment and maintenance of clinics for the use of panel practitioners. The fund is to be administered by Insurance Committees. It would seem that the voluntary hospitals, which in point of fact have been treating insured persons in cases of serious illness, should be accorded recognition for such services in future out of the grant in question.

The remaining items in the foregoing list do not call for special comment, except, perhaps, that it may be pointed out that they are welcome additions to the funds for making health insurance really effective.

### Grants to be Administered by the L.G.B.

There are two further grants included in the estimates which must be mentioned—namely, a sum of £12,000 towards the expenses of local authorities or other bodies in respect of institutions or other provision for maternity and child welfare, and a sum of £50,000 for the provision of laboratory facilities with a view to the prevention, diagnosis, and treatment of disease. These grants are to be administered by the Local Government Board, and it will at once be seen that the purposes for which the money has been voted have a very close connection with the health and well-being of the nation.

